# Targeted Sequencing of Genomic Repeat Regions Detects Circulating Cell-free *Echinococcus* DNA

**DOI:** 10.1371/journal.pntd.0008147

**Published:** 2020-03-10

**Authors:** Zhengqing Wan, Xiaoqing Peng, Lu Ma, Qingshan Tian, Shizheng Wu, Junqi Li, Jie Ling, Weigang Lv, Binrong Ding, Jieqiong Tan, Zhuohua Zhang

**Affiliations:** 1 Institute of Molecular Precision Medicine, Xiangya Hospital, Key Laboratory of Molecular Precision Medicine of Hunan Province, Central South University, Changsha, Hunan, China; 2 Center for Medical Genetics, Central South University, Changsha, Hunan, China; 3 School of Biological and Chemical Sciences, Queen Mary University of London, London, United Kingdom; 4 Center for Prevention and Treatment of Echinococcosis, Qinghai Provincial People's Hospital, Xining, Qinghai, China; 5 Department of Neurology, Qinghai Provincial People's Hospital, Xining, Qinghai, China; 6 Sunrain Biotechnology Corporation, Changsha, Hunan, China; 7 Department of Neurosciences, Hengyang Medical School, University of South China, Hengyang, Hunan, China; University of Würzburg, GERMANY

## Abstract

**Background:**

Echinococcosis is a chronic zoonosis caused by tapeworms of the genus *Echinococcus*. Treatment of the disease is often expensive and complicated, sometimes requiring extensive surgery. Ultrasonographic imaging is currently the main technique for diagnosis, while immunological analysis provides additional information. Confirmation still needs pathological analysis. However, these diagnostic techniques generally detect infection in late stages of the disease. An accurate, early and non-invasive molecular diagnostic method is still unavailable.

**Methodology/Principal findings:**

We sequenced the cell-free DNA (cfDNA) from plasma of echinococcosis patients and confirmed the presence of *Echinococcus* DNA. To improve detection sensitivity, we developed a method based on targeted next-generation sequencing of repeat regions. Simulation experiments demonstrate that the targeted sequencing is sensitive enough to detect as little as 0.1% of an *Echinococcus* genome in 1 mL of plasma. Results obtained using patient plasma shows that the Area Under the Curve (AUC) of the method is 0.862, with a detection sensitivity of 62.50% and specificity of 100%, corresponding to a Youden-index of 0.625.

**Conclusions/Significance:**

This study provides evidence that hydatid cysts release cfDNA fragments into patient plasma. Using the repeat region targeted sequencing method, highly specific detection of *Echinococcus* infection was achieved. This study paves a new avenue for potential non-invasive screening and diagnosis of echinococcosis.

## Author summary

Echinococcosis is a severe chronic parasitic disease caused by tapeworms of the genus *Echinococcus*. According to the World Health Organization, there are more than 1 million people living with echinococcosis worldwide. For decades, little progress has been made to develop a molecular diagnosis and specific treatment for the disease. Although imaging and immunological detection are used for diagnosis, these technologies are either only effective for late stages of the disease or hardly conclusive. The detection of cell-free DNA has been a powerful tool for precise diagnosis. In this study, we showed the presence of *Echinococcus*-derived cell-free DNA in plasma of echinococcosis patients. We further established an assay to detect parasite DNA in blood samples based on amplification of *Echinococcus* specific repeat regions followed by targeted next-generation sequencing. This technique provides a new method for potential extensive screening and precision diagnosis of echinococcosis with high specificity.

## Introduction

Echinococcosis is a severe parasitic disease that predominantly affects agricultural and pastoral areas, especially in South America, Africa, and Asia [1, 2]. The etiological agent of echinococcosis are tapeworms of the genus *Echinococcus* [3]. There are mainly two types of the disease spread in the world, cystic echinococcosis (CE) caused by dog-transmitted *E*. *granulosus* and alveolar echinococcosis (AE) caused by fox-transmitted *E*. *multilocularis* [2]. Humans are accidental intermediate hosts in the life cycle of *Echinococcus*. CE, also known as hydatid disease, is the most common form all over the world. Once an individual is infected by *E*. *granulosus*, the larvae of the tapeworm develop into fluid-filled cysts in various organs [4]. As cysts grow larger over a period of time, symptoms caused by compression will arise, and permanent damage to affected organs will occur [2, 5]. In contrast, the larvae of *E*. *multilocularis* invade in a cancer-like manner to damage the infected organs by infiltrating surrounding tissues, sometimes even metastasizing to other organs.

Clinical symptoms of echinococcosis are atypical and often do not occur until late stages of the disease. Consequently, diagnosis of the disease, especially early diagnosis, is challenging. Ultrasonographic imaging, with the advantage of low cost and rapid diagnostic abilities, is most commonly used [3, 6, 7]. Other imaging techniques with higher resolution such as CT and MRI are employed to detect lesions in specific anatomical locations or atypical echinococcosis [7]. The disadvantage of the imaging techniques is difficult to distinguish *Echinococcus* cysts from other types of cysts. In addition, imaging analysis requires relatively large cysts in CE patients that are already in late stages of the disease [8]. Serological tests may enable earlier diagnosis than imaging technology [9]. Several immunological methods to detect anti-*Echinococcus* antibodies have been developed [9]. However, sensitivity and specificity of these immunological assays vary in different conditions, especially in the cases of CE [10–17]. Therefore, immunological tests are generally used in combination with imaging techniques. Pathological examination of biopsy samples before surgery can be dangerous due to the regeneration capacity of the protoscolex and the risk of anaphylactic reaction during the biopsy procedure [7, 8, 18]. Thus, there is an urgent need for diagnostic methodology that can detect *Echinococcus* infection etiologically and non-invasively.

Assays based on detecting *Echinococcus*-derived circulating antigens were also developed and reported to have high specificity. Unfortunately, their sensitivity was relatively low [17, 19–24]. Consequently, they are barely used in clinic. Likewise, detection of *Echinococcus* DNA by PCR, qPCR or LAMP (loop-mediated isothermal amplification) was also reported [25–31]. These assays are only used for evaluating *Echinococcus* prevalence in dog or fox feces [25, 26, 28, 29, 31] or *Echinococcus* genotyping [27, 30]. Chaya et al. recently reported diagnosis of hydatid disease in humans using PCR detection of parasite DNA in patient serum samples. However, only 25% of hydatid patients appeared positive. All these positive samples had a ruptured cyst confirmed by surgery [32]. No *Echinococcus* DNA was detected in the patient’s urine samples [32]. To the best of our knowledge, no reliable molecular detection method is currently used for non-invasive clinical diagnosis of echinococcosis.

Cell-free DNA (cfDNA) refers to extracellular DNA in various body fluids and its detection in secretions such as blood, urine, and saliva, has been received much attention. The main features of cfDNA include fragmentation, low abundance and fast degradation. The size distribution of fragment length of cfDNA is around 166bp that is close to the length of DNA wrapping around a nucleosome [33, 34]. The concentration of cfDNA is 1~10 ng per 1 mL of human plasma under normal circumstances [35]. It increases up to hundreds of nanograms after exercise [36] or under certain disease conditions [37–39]. The half-life of cfDNA in the free state is about 15 min, but becomes more stable when bound to proteins [34, 37, 39]. cfDNA is commonly released during apoptosis and necrosis of cells from different tissues [40, 41]. High-throughput sequencing can be used to analyze the source of cfDNA, therefore, allowing the non-invasive, safe and accurate real-time monitoring of the primary lesions. Also known as “liquid biopsy” [40], cfDNA detection is widely used in prenatal diagnosis [41–43], early tumor detection [44–46] and organ transplant monitoring [47–50]. Methods to detect diseases involving cell death in specific tissues based on cfDNA methylation patterns are also developed [34, 44, 51–53].

Theoretically, DNA fragments of any foreign cells or organisms can be released into host blood, making it possible to be quickly and accurately detected by high-throughput sequencing [54–56]. In the present work, we demonstrate the presence of *Echinococcus*-derived cfDNA in blood plasma of both CE and AE patients. We further established an assay to detect the parasite DNA based on amplification of repeat regions followed by targeted next-generation sequencing (NGS). This technique opens a possibility to extensively screen and diagnose echinococcosis with high specificity and efficiency.

## Materials and methods

### Sample collection

Plasma and hydatid cyst fluid (HCF) samples from echinococcosis patients were collected from Qinghai province and Xinjiang province in northwest China. All patients fulfilled the diagnostic criteria for echinococcosis [57]. 21 out of 24 patients reported in this study were pathologically confirmed after their blood samples were collected. Three patients (E07, E15 and E20), who had previously diagnosed as echinococcosis and surgically removed cysts, did not take a surgery this time for unwillingness. However, they fitted the diagnostic criteria of Probable cases (E07 and E20) or Possible case (E15) according to WHO-IWGE. CE patients were mainly at stage CE1 or CE2 according to the WHO-IWGE classification. AE patients were at stage P1N0M0 or P2N0M0. For all patients recruited to this study, no chemotherapy was administrated before collecting blood samples. Patients with a sign of either cyst rupture, secondary infection, consolidation or calcification were excluded from the study. Control samples without *Echinococcus* infection and non-relevant control samples with *Schistosoma* infection were collected from Hunan province of central China. The study was approved by the Ethics Committee of Xiangya Hospital of Central South University (Approval No: 201610049). All participants signed a written informed consent. The Standards for Reporting of Diagnostic Accuracy (STARD) checklist and participants flow chart are provided in S1 Checklist and S1 Fig, respectively.

For plasma samples, 10 mL whole blood was collected from each patient and control individual using Streck cfDNA blood collection tubes (218997, Streck, USA). The blood was centrifuged at 1600 g for 10 min at 4°C. The plasma was transferred to a fresh 1.5 mL tube, followed by centrifuging at 16,000 g for 10 min at 4°C to remove remaining cell debris. The plasma was used for further cfDNA extraction. For HCF samples, five intact unilocular hydatid cysts were surgically removed from five unrelated cystic echinococcosis patients. After washing with saline, 3~4 mL HCF was carefully aspirated from each cyst. HCF samples were centrifuged at 16,000 g for 10 min at 4°C. Supernatants were transferred to fresh tubes for further cfDNA extraction. Samples comprising 3 mL of plasma or HCF were used to extract cfDNA using the QIAGEN circulating nucleic acid extraction kit (55114, QIAGEN, Germany) according to the manufacture’s protocol.

### Untargeted cfDNA sequencing

30 ng cfDNA from plasma or HCF was used to prepare NGS libraries with standard protocol and reagents from NEBNext ultra II library kit (E7645, NEB, USA). Due to short length of cfDNA fragments, no further fragmentation was performed. Libraries were then sequenced by Illumina HiSeq X. 35M~50M reads were generated using the PE150 sequencing strategy. Raw data was first processed using trim-galore (Version 0.4.4) in Paired-end mode with default parameters to cut adapter sequences and remove short (< 20bp) or low-quality (Phred score < 20) reads. Duplicated reads were then removed using FastUniq (Version 1.1). For plasma samples, the de-duplicated reads were first mapped to a human reference genome (version hg38) using the bwa-mem algorithm with default parameters. Unmapped reads were extracted using SAMtools followed by searching against the NCBI nt database that includes 43,107,468 sequences in total (last updated in Jun 2017) using BLAST (Basic Local Alignment Search Tool, command line version 2.6.0+). The identity cut-off was 95%. Reads uniquely assigned to *E*. *granulosus* or *E*. *multilocularis* by BLAST were counted. In a “remapping strategy”, no-hit reads from the BLAST analysis were collected and mapped to reference genomes of *E*. *granulosus* (ASM52419v1) or *E*. *multilocularis* (EMULTI002) using the bwa-mem algorithm with default parameters. For HCF samples, data was preprocessed as described above followed by mapping to a reference genome of *E*. *granulosus* (ASM52419v1) using bwa-mem. Unmapped reads were extracted and aligned to a human reference genome (version hg38).

### Identification of repeat sequences of *E*. *granulosus* and *E*. *multilocularis*

Repeat sequences were identified using the RepeatExplorer web server on the Galaxy platform following instruction provided in the on-line manual (http://repeatexplorer.org/) [58]. *E*. *granulosus* and *E*. *multilocularis* sequencing data (ERR112220 and ERR065034 respectively) downloaded from the SRA databases were used as input sequences. High copy number hits were searched using > 0.01% genome proportion as the criterion. To confirm sequence specificity, each unique high copy number sequence was analyzed using BLAST (command line version 2.6.0+). Repeat sequence hits on species other than *Echinococcus spp*. in the NCBI nt database with a percent of identity > 95% were removed. *Echinococcus* specific repeat sequences with lengths between 70 and 2000bp were selected. These filtered sequences were then used for further primer design.

### Primer design and validation

Primers were designed based on identified highly repeated sequences using an on-line primer design tool in NCBI (https://www.ncbi.nlm.nih.gov/tools/primer-blast/). One or two primer pairs for each repeat sequences were designed with product sizes restricted to 70~100bp and Tm of 60 ± 1°C. Primer specificity was checked in the nr/nt database to exclude those likely to produce unintended products.

Each pair of primers was first validated by conventional PCR with a reaction mixture containing 10 μL of Takara Premix EX Taq (RR030A, Takara, Japan), 1 μL primers with a concentration of 10 μmol/L, 100 ng of human genomic DNA with or without 1pg of HCF DNA for the test and control group, respectively. The total reaction volume was brought to 20 μl using ultrapure water (10977023, Invitrogen, USA). The background human DNA was mixed human genomic DNA extracted from whole blood of 5 different individuals. The human genomic DNA was fragmented to size of approximately 160bp using the S2 Focused-ultrasonicator (Covaris, USA). For tests with a reduced amount of template DNA, 100 fg of HCF DNA combined with 10 ng of fragmented human genomic DNA was used as PCR templates. The PCR reaction was performed with an initial denaturation at 95°C for 5 min, followed by 30 cycles of 30 seconds at 95°C for denaturation, 30 seconds at 58°C for annealing and 15 seconds at 72°C for extension. The PCR products of each pair of primers were analyzed on a 2100 bioanalyzer using an Agilent DNA 1000 kit (5067–1504, Agilent, USA). Primer pairs that yielded the intended products were chosen to assemble a multiplex EcDNA (*Echinococcus* cfDNA) primer panel.

The EcDNA primer panel was further verified using HCF DNA samples. The total concentration of the primer panel was 20 μmol/L, with an equal molarity of each primer. Multiplex PCR reactions were performed in a total volume of 20 μL containing 2 μL of the primer panel, 1 μL of 10 ng/μL human genomic DNA, 6 μL of ultrapure water and 1μL of 1 pg/μL HCF DNA and 10 μL of Takara Premix EX Taq DNA polymerase. To increase amplification efficiency, 10 additional touch-down cycles were added prior to aforementioned 30 cycles of amplification, by decreasing the annealing temperature 1°C per cycle from 68°C to 58°C. The multiplex PCR products were purified and subjected to NGS sequencing to confirm the sensitivity of each primer pair.

### Analysis of patient cfDNA samples

The validated primer panel was used to detect *Echinococcus*-derived DNA in patient plasma cfDNA samples. The multiplex PCR reaction mixtures were composed of 10 μL of Takara Premix EX Taq, 2 μL of the EcDNA multiplex primer panel (20 μmol/L), and 8 μL of the circulating cfDNA sample from patients or controls. All samples were renamed before the multiplex PCR procedure for blind evaluation. Reactions were performed using the multiplex PCR conditions with touch down cycles as described above.

The multiplex PCR products were purified using 50 μL of AMPure XP beads (A63881, Beckman Coulter, USA) according to the manufacturer’s protocol. To increase recovery efficiency of short products, 5 μL of PEG buffer consisting of 40% PEG8000 and 10 mM EDTA was added to cleanup reactions. For each sample, 10 ng of recovered DNA was used to construct an NGS library using the NEBNext ultra II library prep kit (E7645, NEB, USA) according to the standard protocol. Libraries were then sequenced on an Illumina MiSeq 500 platform (Illumina, USA) in PE75 mode. An average of 2 M reads was generated for each sample.

The raw sequencing data was initially processed using trim-galore as described above. Clean reads were analyzed using an in-house Perl script (https://github.com/wanzhq/Echinococcus_detection). Briefly, clean data was initially mapped to the hg19 human reference genome using bowtie2. Unmapped reads were extracted and aligned to each intended PCR products or repeat sequences. The mapping ratio was calculated as the proportion of reads that mapped to *Echinococcus* repeats divided by the total number of reads unmapped to hg19.

### Copy number calculation of repeat sequences

The copy numbers of three enriched repeats (Egs-1, Egs-2, Egs-3) were calculated using droplet digital PCR (ddPCR). 5 pg of HCF DNA was used as template for each ddPCR reaction. DdPCRs were done using a Bio-Rad QX200 system (Bio-Rad, USA). A pair of primers specifically amplifying glyoxylate reductase/hydroxypyruvate reductase (EGR-05219) of *E*. *granulosus* genome (forward: GTGTCTTCAACGACGAGGTTAG; reverse: GTCAGCGTAACCATGCAAATG) was included as control. The copy numbers of repeats were normalized to the genome copies of EGR-05219 in the same reaction.

### Statistical analysis

Mapping ratio of unique group were calculated and compared. Data is presented as means ± SEM. The unpaired t-test with Welch's correction was used to calculate p values. Statistical significance was shown as *p < 0.05, **p < 0.01, ***p < 0.001, and ****p < 0.0001. ROC analysis was performed using SPSS statistical software version 16.0 (IBM Corp., USA).

## Results

### Detection of *Echinococcus* DNA in plasma of echinococcosis patients and human DNA fragments in hydatid cyst fluids

To examine whether *Echinococcus* DNA is present in patient plasma samples, we initially performed direct NGS analysis of circulating cfDNA from plasma samples of 16 patients and 11 healthy controls. For each sample, sequencing data was first mapped to the human genome (hg38). Subsequently, unmapped reads were extracted followed by searching against the NCBI nt database using the BLAST algorithm. Reads uniquely aligned to *E*. *granulosus* or *E*. *multilocularis* were considered as *Echinococcus*-derived sequences. As shown in Table 1, DNA of *E*. *granulosus* or *E*. *multilocularis* was detected in 5 out of 16 patient samples, indicating that *Echinococcus* DNA was able to release from hydatid cysts during an infection. Considering the low coverage of the *Echinococcus* genome sequences in the nt database, we collected reads that did not generate hits in the BLAST analysis followed by remapping these reads to the genome of *E*. *granulosus* (ASM52419v1) or *E*. *multilocularis* (EMULTI002) using bwa-mem algorithm. These remapped reads can be regarded as a collection of sequences most likely derived from the *Echinococcus* genome. The ratio of remapped reads in patients was significantly higher than that in controls (S2 Fig). *Echinococcus* DNA was detected in 9 out of 16 patients with remapping ratio above 100×10^−6^ (Table 1). For AE, parasite-derived DNA was detected in 4 out of 5 patients.

**Table 1:** BLAST analysis and realignment of plasma cfDNA of echinococcosis patients and controls.

Group	Samples	Mapped to Human Genome	Reads Unmapped to Human Genome	Unique BLAST hits for E. g or E. m	Reads without BLAST hits	Reads Mapped to E. g or E. m	Ratio to E. g or E. m (×10^−6^)
Cystic Echinococcosis Patients	E01	98.78%	666337	0	362558	31	85.504
	E02	98.14%	987872	0	584146	80	136.952
	E03	97.82%	789692	0	461545	261	565.492
	E05	98.14%	884296	0	511312	78	152.549
	E06	98.92%	461088	0	302465	6	19.837
	E09	98.57%	592295	0	448111	8	17.853
	E16	98.89%	1698688	0	1417945	91	64.177
	E17	99.55%	138359	0	117844	0	0.000
	E18	99.31%	203737	2	158564	54	340.556
	E19	99.73%	186700	0	94623	29	306.479
	E20	98.65%	507440	0	373063	9	24.125
Alveolar Echinococcosis Patients	E07	99.30%	389896	2	290186	205	706.443
	E08	98.98%	456714	6	346458	101	291.522
	E10	99.23%	382636	0	328750	12	36.502
	E11	99.20%	367624	32	259077	1173	4527.611
	E12	99.27%	344414	21	248630	698	2807.384
Normal Controls	CT01	99.31%	473305	0	327226	4	12.224
	CT02	99.24%	431979	0	265245	2	7.540
	CT03	98.59%	884409	0	813749	23	28.264
	CT04	99.30%	212610	0	186636	6	32.148
	CT05	99.35%	189895	0	171294	3	17.514
	CT06	98.65%	708331	0	378457	24	63.415
	CT07	98.68%	618738	0	504094	29	57.529
Schistosomiasis Patients	S01	99.32%	224216	0	180962	5	27.630
	S02	99.43%	215004	0	176148	5	28.385
	S03	99.56%	154415	0	133285	2	15.005
	S04	99.46%	154279	0	129377	0	0.000

E. g: *E*. *granulosus*. E. m: *E*. *multilocularis*. BLAST: NCBI Basic Local Alignment Search Tool. Unique BLAST hits for E. g or E. m: number of reads that were only matched to E. g or E. m sequences in the NCBI nt database. Reads without BLAST hits: number of reads that did not match any sequences in the NCBI nt database. Reads Mapped to E. g or E. m: number of reads that did not generate BLAST hits but could be mapped to the genome of E. g or E. m using bwa-mem algorithm. Ratio to E. g or E. m: mapping ratio of the mapped reads set divided by the set of reads without BLAST hits.

**Fig 1:**
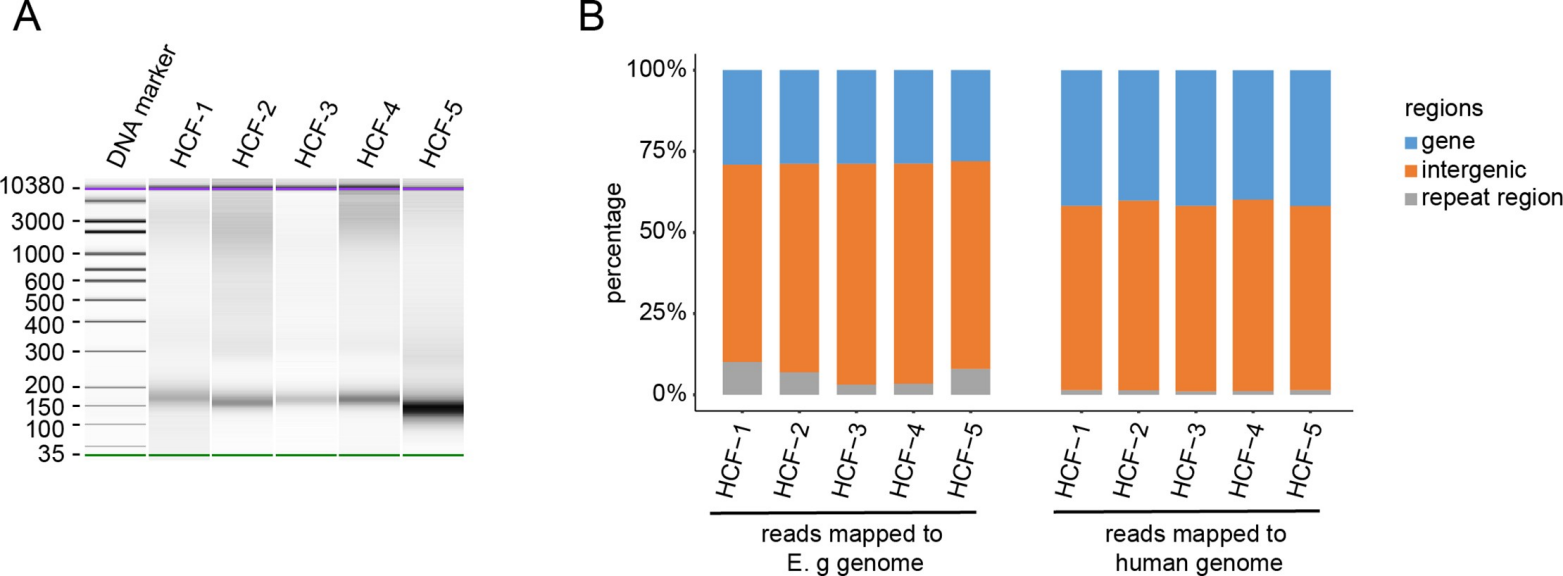
Profiles of cfDNA extracted from intact hydatid cysts. (A) Size of cfDNA from HCF samples analyzed on a 2100 bioanalyzer. Molecular weight (bp) is indicated (left lane). Green lines:15bp. Purple lines: 1500bp. (B) Profile of the cfDNA sequencing reads mapped to the *E*. *granulosus* genome (ASM52419v1) and human genome (hg38).

To further verify the wall of hydatid cysts could allow DNA fragments to pass through, we extracted cfDNA from the HCF of intact hydatid cysts from 5 unrelated patients followed by NGS. Despite limited contamination with large fragments of genomic DNA, predominant size of cfDNA from HCF was approximately the same as that of human plasma cfDNA (Fig 1A). Sequencing reads mapped to the human reference genome were detected in all 5 HCF samples (Table 2). The proportion of human-derived DNA sequences reached as high as 80% of the total cfDNA sequencing reads. The lowest ratio of reads within one cyst that matched to the human genome was about 12%. Results suggest that the hydatid cyst wall is permeable at least to short, freely circulating nucleic acids, such as cfDNA. In these cases, further mapping analysis revealed that most of the identified sequences of *E*. *granulosus* cfDNA were from non-coding, low complexity regions. In contrast, human cfDNA sequences were from both coding and non-coding regions of the genome (Fig 1B).

**Table 2:** Alignment results of the reads obtained from HCF sequencing.

Samples	Total Reads	Reads Mapped to the E. g Genome	Percentage of Reads Mapped to E. g genome	Reads Mapped to the Human Genome	Percentage of Reads Mapped to Human genome
HCF-1	50975739	7216672	14.16%	43343421	85.03%
HCF-2	15738707	3723808	23.66%	11924502	75.77%
HCF-3	15827757	12114428	76.54%	2883866	18.22%
HCF-4	15325904	12961579	84.57%	1863580	12.16%
HCF-5	33656866	6281636	18.66%	27331719	81.21%

HCF: hydatid cyst fluid; E. g: *E*. *granulosus*.

### Multiplex PCR panel design

To increase detection sensitivity of cfDNA at trace levels, we employed a targeted multiplex PCR of repeat regions to enrich *Echinococcus*-derived cfDNA before NGS. A total of 19732 repeat sequences were identified using RepeatExplore on the Galaxy platform (S1 Appendix), based on *E*. *granulosus* (ERR112220) and *E*. *multilocularis* (ERR065034) sequences downloaded from the NCBI SRA database. To improve the template utilization efficiency, length of products was restricted to 70~100bp considering ~166bp of typical cfDNA. A total of 16 primer pairs were initially selected for validation (Table 3).

**Table 3:** Primers selected and their target repeat sequences.

Primer Pairs	Repeat Names	Forward Primer	Reverse Primer	Minimal Product Length (bp)**
Egs-1	>CL11Contig2	CACTGTGACGTCATCTGGCCT	TCAGGTGACGTAATGGAGGGTCT	70
Egs-2	>CL22Contig1	TCTGCTGCTTGCATTCACAC	CAAATGCTCGGTACACCACG	84
Egs-3	>CL34Contig1	CTGCAATAGCACCCAATTCACA	GAAGGAGTATCGTTGGTACGCT	86
mgs-1	>CL5Contig3	TTCGTGCTACGACTTTCTCCAC	GGAGTGCAAATGAAGTAGATGCG	72
mgs-2	>CL10Contig9	TCAAGTATGTTGCGAAGGCGA	TGCATGGTAGAGACCCGGAA	76
mgs-3	>CL13Contig14	GTTGCCAGGGCAGTGAGTTA	TGGCATTGGGCGTGAAGTAG	71
mgs-4	>CL17Contig31	AGTAGCGGAACGGTGGATTT	ACAATGGCCGGTAGTGAAGA	83
mgs-5	>CL19Contig16	CCACCCAGCGAGGTACAAG	AGTGGTTTATCCCTCGGTTCTG	75
mgs-6	>CL21Contig4	GTTTCACACCGACAACTGCAA	TGAGTCGAAGGCGAACACC	76
mgs-7	>CL23Contig4	GGATCCGTCGATGTTAGCGT	CCGCCATAGAGGGTAGATGC	71
mgs-8	>CL10Contig9	ATCCGCACCTGTCGTAACTT	GTAACATGCGGCTTCGGAAT	88
mgs-9	>CL13Contig14	CGGGACACATCCTAACTGGT	CCGGTCATCCATGGGGATTG	83
mgs-10	>CL23Contig4	CAGAGGCTCGTTTGTGGTCA	GGTGCACATTAAATACCAAAACCC	73
mgs-11	>CL26Contig5	CGAAGGACAGCCATTTCGGA	TCAGCGAGCCACAGATTACAT	70
mgs-12	>CL27Contig3	TGGCGCAACACCTTGTAGAT	GAAGGTGAAGGTGCCGAAGA	87
mgs-13	>CL29Contig14	homology to human histone gene*		/
mgs-14	>CL30Contig2	AGCACTCCTCATCAGTCAACTC	CTGAAACATGCTAAAGGTATGCGT	82

* Repeat sequence >CL29Contig14 was excluded because it is highly homologous to human histone genes.

** Size of actual products may be different from predicted due to the possibility of tandem repeat and indels in repeats.

**Fig 2:**
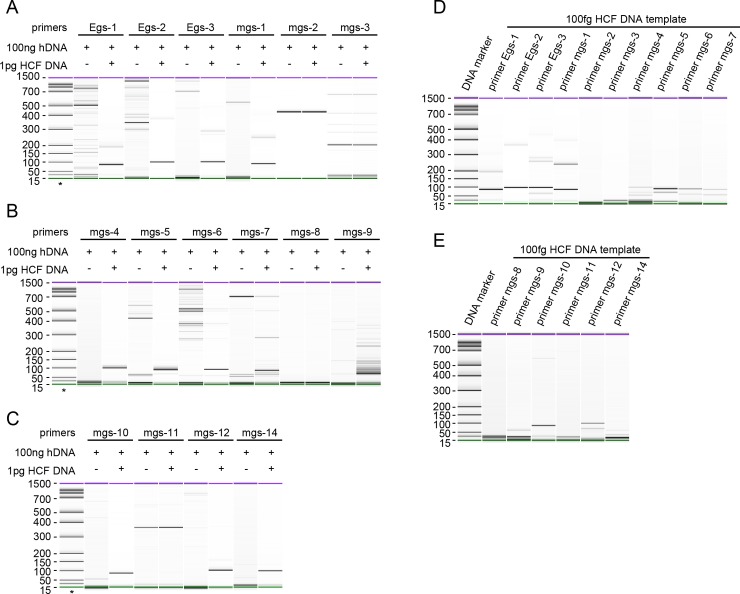
Validation of primers via conventional PCRs. (A, B, C) Conventional PCR amplification with 1 pg of HCF DNA as template in the background of 100 ng of human genomic DNA with each primer pair. Expected products were observed with the primer pairs Egs-1, Egs-2, Egs-3, mgs-1, mgs-4, mgs-5, mgs-6, mgs-7, mgs-9, mgs-10, mgs-12 and mgs-14. (D, E) PCR sensitivity analysis by decreasing the HCF DNA template to 100 fg. Products of the expected size were observed with 10 primer pairs. The molecular weight (bp) is indicated on the left of each panel. * indicates DNA marker. Green lines:15bp, Purple lines: 1500bp.

**Fig 3:**
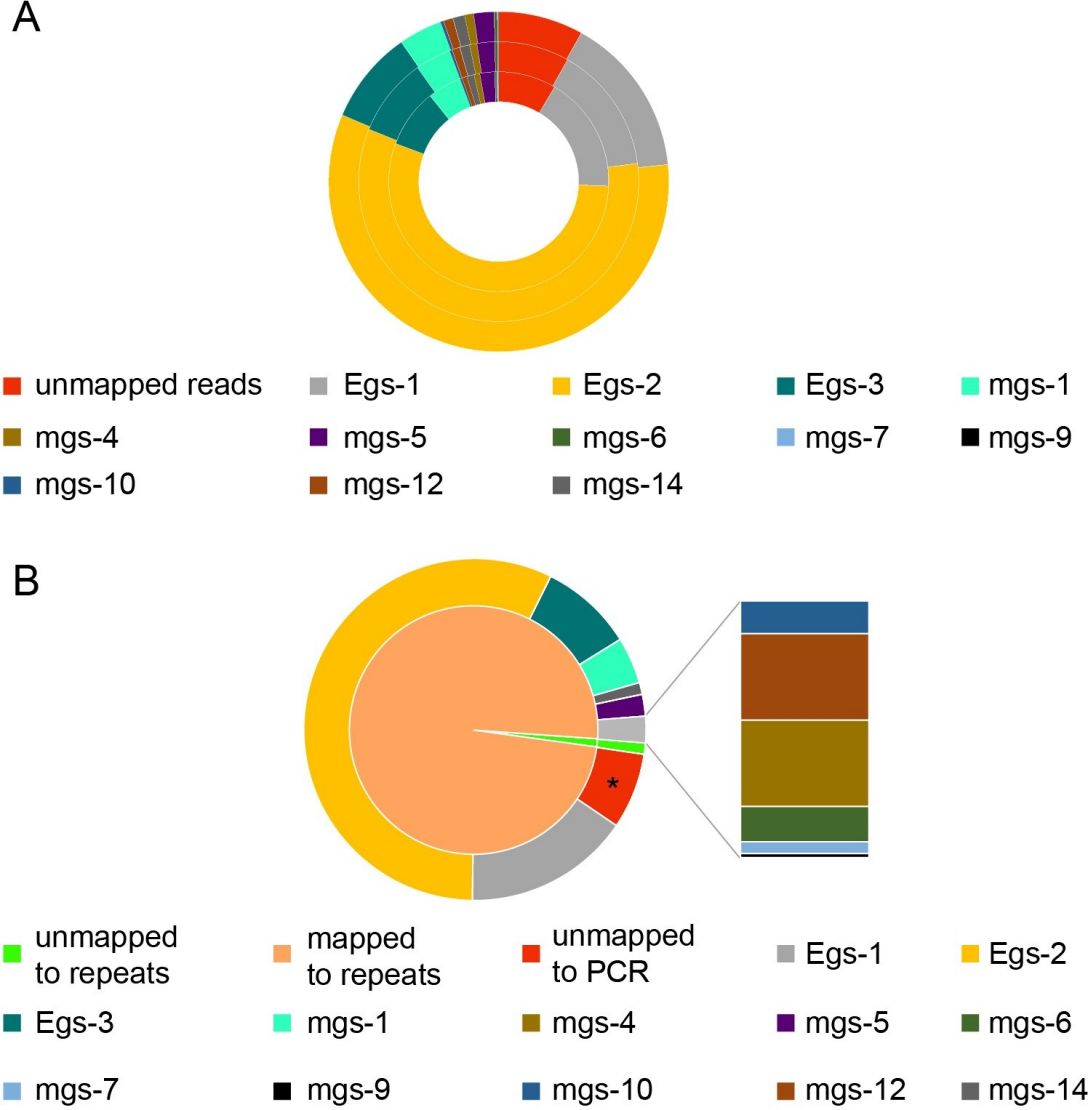
PCR products observed in multiplex PCR. (A) Results of three independent samples are shown. Each pair of primers yielded a product of expected sequence. (B) Comparison of average mapping ratio to repeat sequences and intended products. * Part of the reads unmapped to the intended products were mapped to repeat sequences.

Each pair of candidate primers were validated by conventional PCR using a mixture of HCF-1 HCF cfDNA and human genomic DNA as templates. A total of 12 pairs of primers generated anticipated products with 1 pg HCF cfDNA mixed with 100ng of human genomic DNA. On the other hand, no specific band was detected using human DNA alone as templates (Fig 2A–2C). Further analysis revealed that 10 pairs of primers were sensitive enough to obtain specific PCR products using 100 fg of HCF cfDNA as templates (Fig 2D and 2E). 13 pairs of primers yielded specific PCR products using DNA extracted from the liver lesion of an alveolar echinococcosis patient (S3 Fig). As our primary goal was to detect CE, 12 pairs of primers that gave specific products with HCF DNA were combined into a EcDNA (*Echinococcus* cfDNA) primer panel. Multiplex PCR followed by NGS showed that the primer panel is effective to amplify *Echinococcus* DNA from 1 pg HCF DNA (Fig 3A, Table 4). Considering that human DNA is present in hydatid cysts, we further analyzed the copy number of *Echinococcus* genome per 100 pg HCF DNA by droplet digital PCR. The results showed that the copy number of the *Echinococcus* genome per 100 pg HCF DNA varied from 20 to 400, which was consistent with results of NGS analysis (Table 2 and S4 Fig). The approximate repeat numbers for Egs-1, Egs-2 and Egs-3 per one *Echinococcus* genome were 7000, 800 and 400, respectively (S4 Fig).

**Table 4:** Validation of the EcDNA primer panel using multiplex PCR followed by NGS.

		1pg-1	1pg-2	1pg-3	Average
Hit for Repeats		98.32%	99.26%	99.22%	98.93%
Hit for PCR		91.51%	91.81%	91.93%	91.75%
Mapped to Each Primer Product					
	Egs-1	17.17%	14.74%	15.34%	15.75%
	Egs-2	54.98%	58.20%	58.02%	57.06%
	Egs-3	8.64%	9.04%	8.88%	8.85%
	mgs-1	4.98%	4.13%	4.12%	4.41%
	mgs-4	0.86%	0.85%	0.83%	0.85%
	mgs-5	2.17%	2.03%	1.94%	2.05%
	mgs-6	0.34%	0.35%	0.32%	0.33%
	mgs-7	0.21%	0.08%	0.08%	0.12%
	mgs-9	0.03%	0.04%	0.04%	0.04%
	mgs-10	0.32%	0.31%	0.31%	0.31%
	mgs-12	0.73%	0.91%	0.91%	0.85%
	mgs-14	1.07%	1.15%	1.14%	1.12%

Hit for Repeats: mapping ratio to the repeat sequences. Hit for PCR: mapping ratio to intended PCR products. 1pg-1, 1pg-2, 1pg-3: three independent experiments using 1pg HCF DNA as multiplex PCR templates.

NGS analysis of multiplex PCR products revealed that the ratio of reads mapped to repeat sequence dataset (98.93% on average) was higher than that of reads exactly mapped to the anticipated PCR products (91.75% on average). The difference likely resulted from the variability of each repeat in genome (Fig 3B, Table 4). Therefore, further analysis was based on the ratio of reads mapped to the repeat regions rather than those mapped to the anticipated PCR products.

### The EcDNA primer panel detect *Echinococcus* DNA using femtogram HCF cfDNA as templates

**Fig 4:**
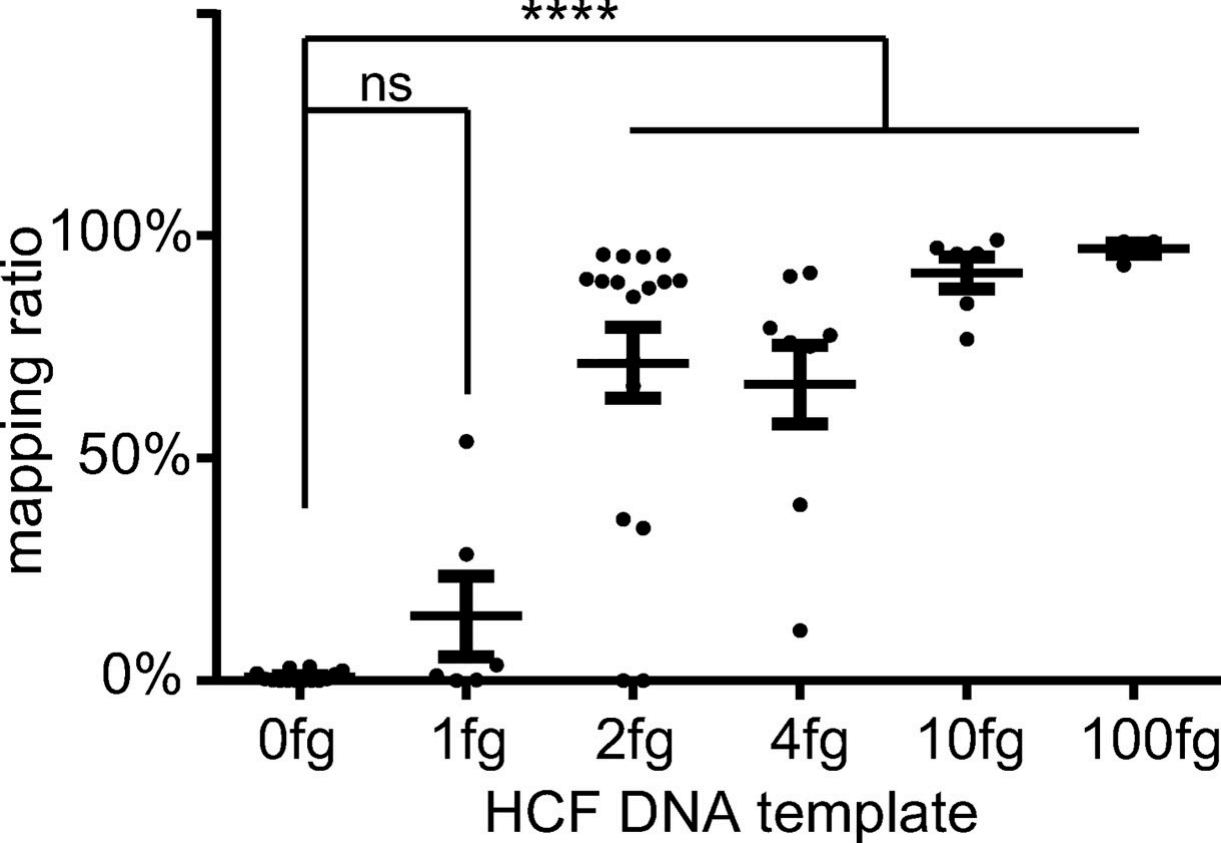
Simulation experiments with various quantities of HCF DNA template. The mapping ratio to the *Echinococcus* genome indicates the ratio of reads mapped to repeat sequences over the total number of reads. Data are expressed as means ± SEM. **** p < 0.0001, unpaired t-test with Welch's correction. Note that detection limit of the EcDNA (*Echinococcus* cfDNA) primer panel was as low as 2 fg/reaction.

To further determine the detection limit of the EcDNA primer panel, we titrated HCF cfDNA templates mixed with human cfDNA. The results showed that the EcDNA panel was sensitive enough to stably detect as little as 2 fg cfDNA from the HCF-1 sample (Fig 4, S1 Table). To further verify the detection sensitivity, we added various quantities of HCF cfDNA to plasma isolated from healthy controls, followed by extracting cfDNA from the mixed plasma. cfDNA isolated from the mixtures were subjected to multiplex PCR and NGS. The results revealed that the detection sensitivity was as little as 5 fg HCF cfDNA per 1 mL of plasma (S2 Table). The quantitative results of ddPCR suggest approximately 30 copies of *Echinococcus* genome per 100 pg of DNA in the HCF-1 sample (S4 Fig). Therefore, the method is sensitive enough to detect 0.1% of *Echinococcus* genome per 1mL of plasma.

### Detection of *Echinococcus* DNA using cfDNA from patient plasma samples

The EcDNA primer panel was next used to analyze cfDNA from plasma samples of patients infected with *Echinococcus* (n = 24). Healthy uninfected individuals (n = 27) and patients infected with *Schistosoma* (n = 9) were also analyzed as controls. A clinical summary of controls and patients is shown in Table 5. Since no method is available to exclude *Echinococcus* infection in early stages, all control samples were collected from Hunan province of central China, where echinococcosis is rarely reported. Further follow-up of the controls finds no infection with *Echinococcus* of these individuals. Detailed information of patients is shown in Table 6.

**Table 5:** Overall information of plasma samples.

Sample Group	Echinococcosis patients (n = 24)	Schistosomiasis patients (n = 9)	Uninfected controls (n = 27)
Age (years)	38.08 ± 2.828	42.78 ± 3.609	36.60 ± 2.261
Gender (male/female)	11/13	5/4	14/13
Imaging-based Examination (positive/negative/not determined)	24/0/0	0/9/0	0/11/16
Immunological Examination (positive/negative/not determined)	18/2/4	0/0/9	0/0/27
Pathological Examination (positive/negative/not determined)	21/0/3	0/0/9	0/0/27

The age was shown as mean ± SEM.

**Table 6:** Detailed clinical information of echinococcosis patients.

Sample ID	Gender	Age(years)	Nationality	Clinical Diagnosis	Previous *Echinococcus* Infection History*	Imaging-based Examination	Immunological Examination	Stages**	Specific Signs	**Pathological Diagnosis**
E01	male	36	Chinese-Tibetan	hepatic cystic echinococcosis	N	P	P	CE1	N	cystic echinococcosis
E02	male	41	Chinese-Han	diaphragmatic cystic echinococcosis	N	P	N	CE3	N	cystic echinococcosis
E03	male	43	Chinese-Tibetan	pelvic and abdominal cystic echinococcosis	N	P	P	ND	N	cystic echinococcosis
E05	female	43	Chinese-Tibetan	diaphragmatic cystic echinococcosis	N	P	P	CE2	N	cystic echinococcosis
E06	female	34	Chinese-Tibetan	abdominal cystic echinococcosis	P	P	P	ND	N	cystic echinococcosis
E07	female	34	Chinese-Tibetan	hepatic alveolar echinococcosis	P	P	P	P2N0M0	N	ND
E08	female	36	Chinese-Tibetan	hepatic alveolar echinococcosis	P	P	P	P1N0M0	N	alveolar echinococcosis
E09	female	29	Chinese-Han	hepatic cystic echinococcosis	P	P	ND	CE1	N	cystic echinococcosis
E10	female	36	Chinese-Tibetan	hepatic alveolar echinococcosis	N	P	P	P1N0M0	N	alveolar echinococcosis
E11	female	20	Chinese-Tibetan	hepatic alveolar echinococcosis	P	P	P	P2N0M0	left hepatic vein, hepatic segment inferior vena cava involvement	alveolar echinococcosis
E12	female	40	Chinese-Tibetan	hepatic alveolar echinococcosis	P	P	P	P2N0M0	right hepatic vein, right portal vein, inferior vena cava involvement	alveolar echinococcosis
E14	male	41	Chinese-Tujia	hepatic cystic echinococcosis	N	P	P	CE2	N	cystic echinococcosis
E15	female	68	Chinese-Tibetan	hepatic cystic echinococcosis	P	P	N	CE2	residual cavity infection	ND
E16	male	27	Chinese-Uighur	hepatic cystic echinococcosis	N	P	ND	CE2	N	cystic echinococcosis
E17	female	37	Chinese-Kazak	hepatic cystic echinococcosis	N	P	ND	CE1	N	cystic echinococcosis
E18	female	30	Chinese-Hui	hepatic cystic echinococcosis	N	P	ND	CE1	N	cystic echinococcosis
E19	male	7	Chinese-Uighur	hepatic cystic echinococcosis	N	P	P	CE1	N	cystic echinococcosis
E20	male	45	Chinese-Uighur	hepatic and abdominal cystic echinococcosis	P	P	P	CE2	sinus formation	ND
E21	male	27	Chinese-Uighur	hepatic cystic echinococcosis	N	P	P	CE1	N	cystic echinococcosis
E22	male	70	Chinese-Han	hepatic cystic echinococcosis	N	P	P	CE1	multiple hydatid cyst	cystic echinococcosis
E23	female	38	Chinese-Kazak	hepatic cystic echinococcosis	N	P	P	CE2	N	cystic echinococcosis
E24	male	63	Chinese-Han	hepatic cystic echinococcosis	N	P	P	CE1	N	cystic echinococcosis
E25	male	33	Chinese-Hui	hepatic cystic echinococcosis	N	P	P	CE2	N	cystic echinococcosis
E26	female	36	Chinese-Uighur	hepatic cystic echinococcosis	N	P	P	CE1	N	cystic echinococcosis

P: positive. N: negative. ND: not determined.

* Infection history with the same type of echinococcosis.

**Stages were determined according to the WHO-IWGE classification.

**Fig 5:**
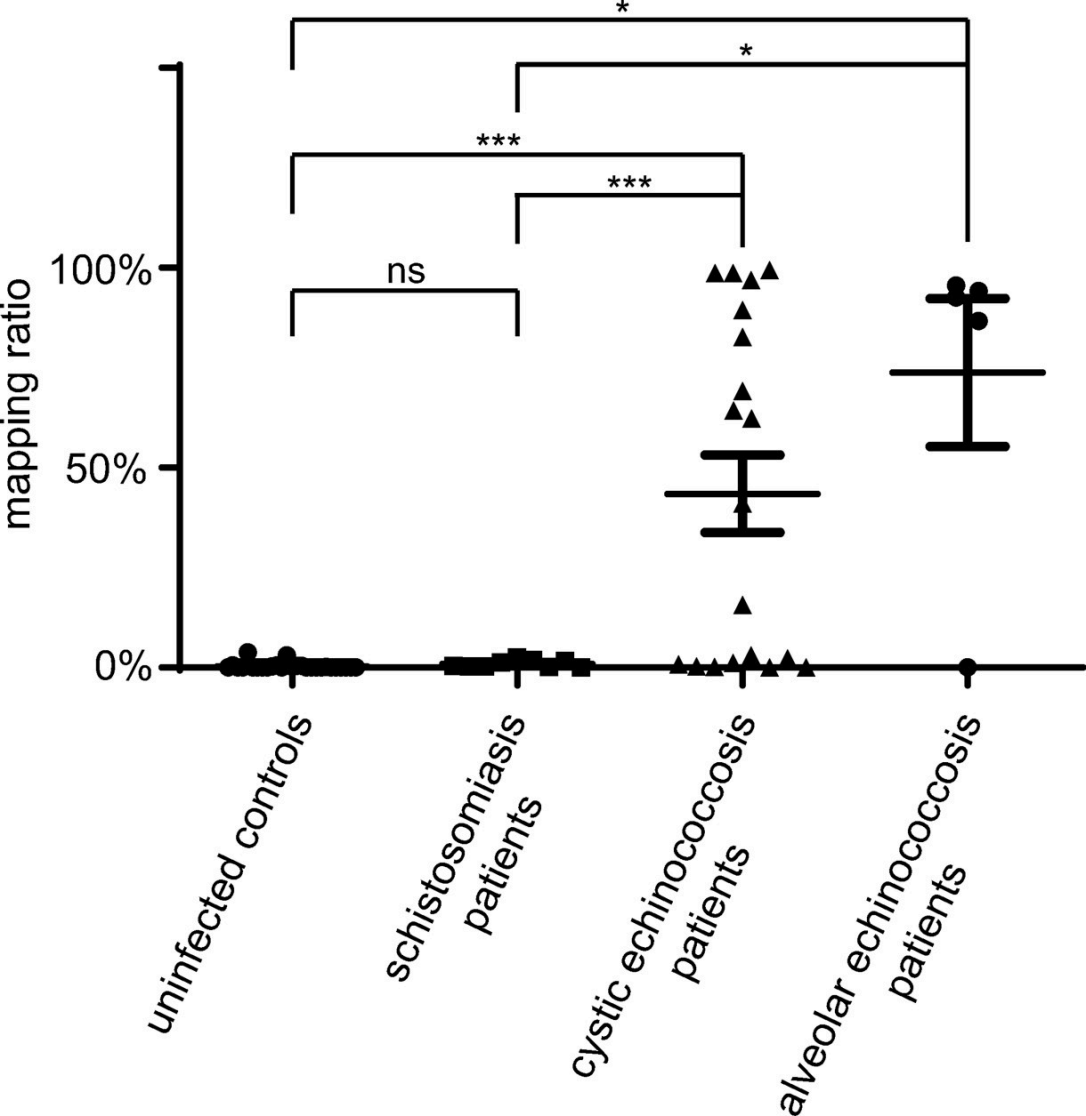
The mapping ratio to the *Echinococcus* genome of plasma cfDNA samples from CE, AE patients and control individuals. Data are expressed as means ± SEM. *** p < 0.001, * p < 0.05, unpaired t-test with Welch's correction.

The results showed that the mapping ratio, calculated as the proportion of reads mapped to the *Echinococcus* DNA repeats over the total number of reads unmapped to the human reference genome, was significantly higher in samples from alveolar and cystic echinococcosis patients than those of schistosomiasis patients or uninfected control individuals (Fig 5). *Echinococcus*-derived cfDNA was detected in 15 out of 24 plasma samples from echinococcosis patients with cut-off to 10.0%. In contrast, no *Echinococcus*-derived DNA was detected in the cfDNA isolated from plasma of schistosomiasis patients and healthy control individuals (Table 7). Results indicate that the targeted detection method is more sensitive than the direct sequencing method.

**Table 7:** Mapping ratio to *Echinococcus* repeat sequences of circulating cfDNA of echinococcosis patients and controls.

Cystic echinococcosis Patients	Mapping Ratio	Alveolar echinococcosis Patients	Mapping Ratio	Uninfected Controls	Mapping Ratio	Schistosomiasis Patients	Mapping Ratio
E01	82.74%	E07	92.56%	CT01	0.52%	S01	0.38%
E02	15.74%	E08	86.66%	CT02	0.03%	S02	1.88%
E03	96.94%	E10	0.01%	CT03	3.84%	S03	0.34%
E05	0.22%	E11	95.62%	CT04	0.38%	S04	0.30%
E06	89.45%	E12	94.13%	CT05	0.06%	S05	1.26%
E09	0.76%			CT06	3.08%	S06	2.51%
E14	41.03%			CT07	0.21%	S07	1.74%
E15	98.67%			CT08	0.32%	S08	0.13%
E16	98.73%			CT09	0.55%	S09	0.02%
E17	0.02%			CT10	0.00%		
E18	99.49%			CT11	0.01%		
E19	69.15%			CT12	0.11%		
E20	62.31%			CT13	0.01%		
E21	2.34%			CT14	0.00%		
E22	64.31%			CT15	0.01%		
E23	1.14%			CT16	0.01%		
E24	3.06%			CT17	0.29%		
E25	0.32%			CT18	0.45%		
E26	0.04%			CT19	0.01%		
				CT20	0.01%		
				CT21	0.01%		
				CT22	0.01%		
				CT23	0.02%		
				CT24	0.03%		
				CT25	0.02%		
				CT26	0.09%		
				CT27	0.04%		

Mapping ratio was calculated as the proportion of reads mapped to the *Echinococcus* DNA repeats over the total number of reads unmapped to the human reference genome.

**Fig 6:**
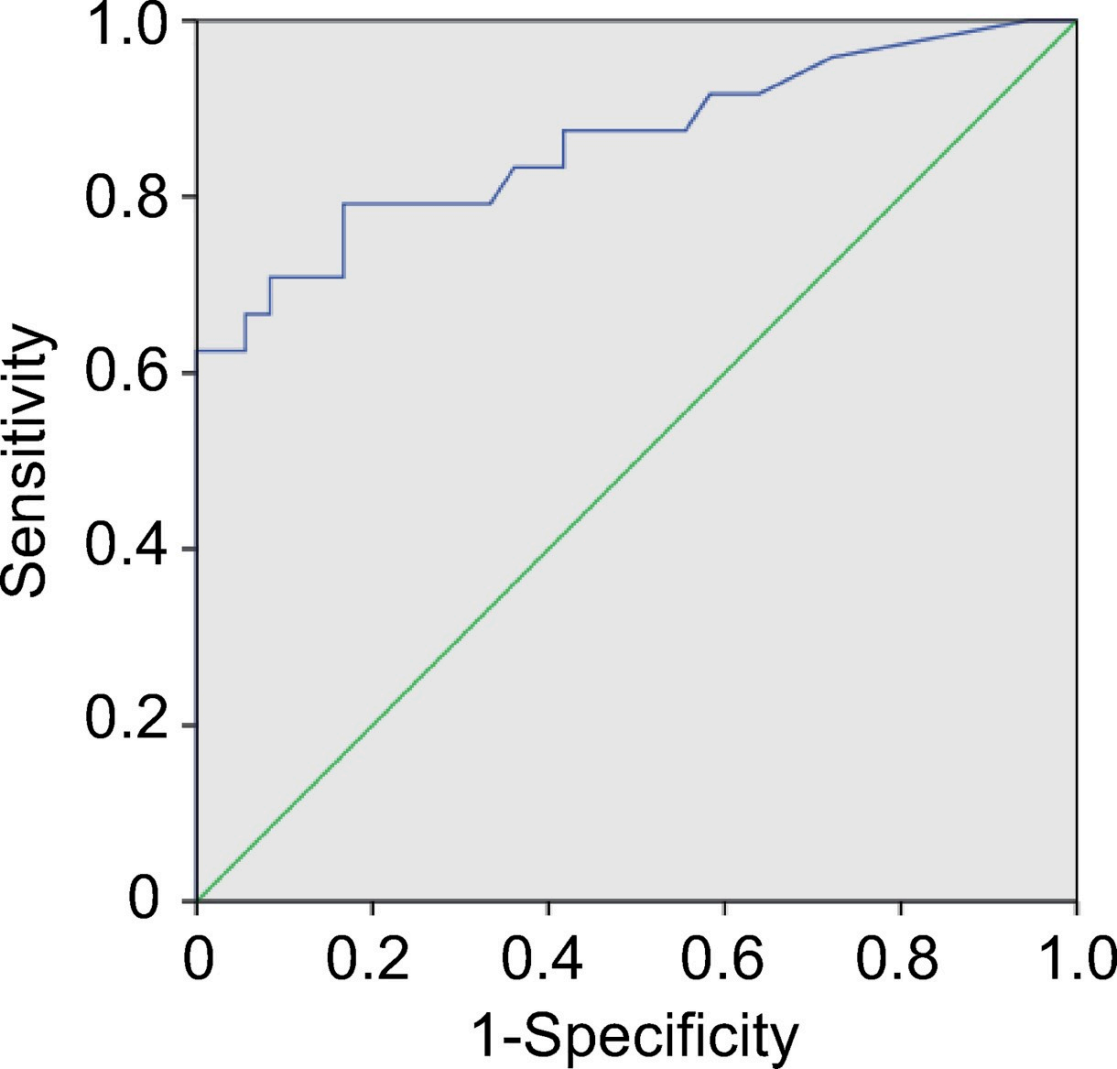
ROC curve based on the mapping ratio of repeat regions with echinococcosis patient samples. Blue line: the ROC curve based on the results of targeted sequencing of repeat regions in this study. Green line: random classification.

A ROC analysis based on the mapping ratio of plasma samples showed that the area under the curve (AUC) was 0.862, with a 95% confidence interval of 0.758~0.965 (Fig 6). When the cut-off was set to 9.79%, the ROC curve reached a Youden-index of 0.625, with a detection sensitivity of 62.5% and specificity of 100%.

## Discussion

*Echinococcus* parasitizes deeply in human organs such as liver, lung and brain. Due to the risk of disseminated infection, it is not advisable to conduct a biopsy to make a definitive diagnosis. Thus, non-invasive methodology for early diagnosis is urgently needed. In theory, hydatid cysts allow nutrients in and metabolites out in order to grow [59]. In this study, we show that *Echinococcus* derived cfDNA is present in plasma of *Echinococcus* infected individuals, which could facilitate non-invasive diagnosis. We further designed a targeted NGS strategy to detect *Echinococcus*-derived cfDNA in blood samples. The results suggest that targeted NGS is feasible to achieve non-invasive molecular diagnosis of echinococcosis.

Targeted NGS detected *Echinococcus* cfDNA in patient plasma in this study. The finding provides the first evidence to our knowledge that *E*. *granulosus* DNA is also released from intact hydatid cysts into blood of infected individuals, laying the basis for a non-invasive and precise diagnosis of echinococcosis using DNA sequencing. It is likely that the identified *Echinococcus* DNA fragments pass through the wall of hydatid cysts to enter the blood stream. This notion is further supported by the discovery of human DNA fragments inside hydatid cysts, despite that patient cfDNA in hydatid cysts is remarkably higher than parasite cfDNA in patient serum.

The concentration of *Echinococcus* cfDNA in patient plasma is likely very low, suggesting that release of *Echinococcus* DNA fragments from hydatid cysts is either limited or that the DNA is rapidly degraded. It is possible that protein-bounded DNA has a limited capacity to pass through the wall of hydatid cysts. The proportion of *Echinococcus* DNA in the cfDNA isolated from patient plasma varies between 1/1000 and 1/100000, which is a major barrier for clinical detection using untargeted NGS. We therefore developed a targeted multiplex PCR primer panel to amplify repeat sequences from the *Echinococcus* genome in order to specifically enrich *Echinococcus*-derived cfDNA for further analysis via NGS. The combination of multiplex PCR and NGS incorporates both the sensitivity of PCR and the specificity of DNA sequencing while avoiding cross-reactions or false positives. This method is sensitive enough to detect a thousandth of an *Echinococcus* genome in simulation experiments. Meanwhile, targeted NGS cut down the sequencing cost from hundreds of dollars for an untargeted sequencing experiment to less than 1 dollar for a targeted sequencing detection. The overall cost for each detection is approximately $50 in laboratory that can be reduced to less than $20 with further improvement.

Several PCR or qPCR methods for the detection of *Echinococcus* DNA in patients have been reported by various research groups [25–31]. However, these methods have not demonstrated non-invasive diagnostic values for echinococcosis. Potential reasons could be diverse. One possibility is the low abundance of *Echinococcus* DNA in patient plasma. Alternatively, DNA extraction methods used in previous studies are generally optimized for genomic DNA extraction that is not optimized for short cfDNA fragments. Furthermore, the designed length of PCR products was usually longer than the average length of cfDNA fragments (166bp), reducing detection of short DNA fragments. A recent study on the detection of parasite-derived cfDNA from serum of alveolar echinococcosis (AE) patients partly improved cfDNA extraction and primer design protocol. These authors were able to distinguish all samples from an AE animal model and 30% of patient serum samples via qPCR and ddPCR [60]. The present study enriched targeted DNA fragments by multiplex PCR followed by NGS, which increased the possibility to detect low abundant and short cfDNA fragments. In the present study, the AUC was 0.862, with a detection sensitivity of 62.50% and specificity of 100%, corresponding to a Youden-index of 0.625. Interestingly, the sensitivity increased to 80% if we consider only the 5 alveolar echinococcosis patients. This is likely because of its distinct way of expansion of *E*. *multilocularis* in host body allowing cfDNA to release to blood stream. The detection sensitivity may be even higher for early infected patients before formation of a complete cyst, because the laminated layer serves as a physiochemical barrier that reduces the permeability of macromolecules [5]. Unfortunately, no technology that diagnose *Echinococcus* infection in very early stages is available. A more comprehensive clinical study with larger numbers of patients will be needed to further verify the effectiveness of the method for early diagnosis.

Our data provides evidence that hydatid cysts release cfDNA into patient plasma even if the cyst membrane remains intact. This study also establishes a new and practical method based on the concept of cell-free DNA in conjunction with the high sensitivity of multiplex PCR and the specificity of NGS, paving a new avenue for a potential early, accurate and non-invasive diagnosis of echinococcosis.

## Supporting information

S1 Checklist Standards for Reporting of Diagnostic Accuracy (STARD) checklist. (DOCX)

S1 Fig Flow diagram of participants enrolled in this study. (TIF)

S2 Fig Remapping ratios of plasma cfDNA from echinococcosis patients and control individuals. Remapping ratio was transformed by logarithm. E17 and S04 were discarded as outliers because their remapping ratio was zero. The data are expressed as mean ± SEM. * p < 0.05, ** p < 0.01, unpaired t-test with Welch's correction.
(TIF)

S3 Fig Test of primer pairs with *E*. *multilocularis* DNA. PCR amplification of 100 pg genomic DNA extracted from liver lesion of an alveolar echinococcosis patient in the background of 10 ng human genomic DNA with each pairs of primers. Products of the expected size were observed with primers Egs-2, mgs-1, mgs-2, mgs-3, mgs-4, mgs-5, mgs-6, mgs-7, mgs-8, mgs-9, mgs-11, mgs-12 and mgs-14. The molecular weight (bp) is indicated on the left of each panel. Green lines:15bp. Purple lines: 1500bp.
(TIF)

S4 Fig Copy number evaluation of *E*. *granulosus* genome and repeat. (A) Copy numbers per 100 pg of *E*. *granulosus* genome from HCF samples are shown. (B) Copy numbers amplified by indicated 3 pair of primers in one *Echinococcus* genome. Data is expressed as mean ± SD.
(TIF)

S1 Table Mapping ratio of sequencing reads from simulation experiments using various quantities of HCF templates. (XLSX)

S2 Table Alignment results of plasma samples added HCF DNA. Indicated quantity of HCF DNA was added per 1mL of plasma from health controls. 5 ng of lambda DNA was added as quality control for cfDNA extraction.
(XLSX)

S1 Appendix All repeat sequences identified using RepeatExplore on the Galaxy platform, based on *E. granulosus* and *E. multilocularis* sequencing data downloaded from NCBI. (FASTA)
