# Supplementary figures and images for: Targeted Sequencing of Genomic Repeat Regions Detects Circulating Cell-free Echinococcus DNA
Source: PLoS Negl Trop Dis. 2020 Mar 10;14(3):e0008147. doi: 10.1371/journal.pntd.0008147 (PMC7083330; doi:10.1371/journal.pntd.0008147)

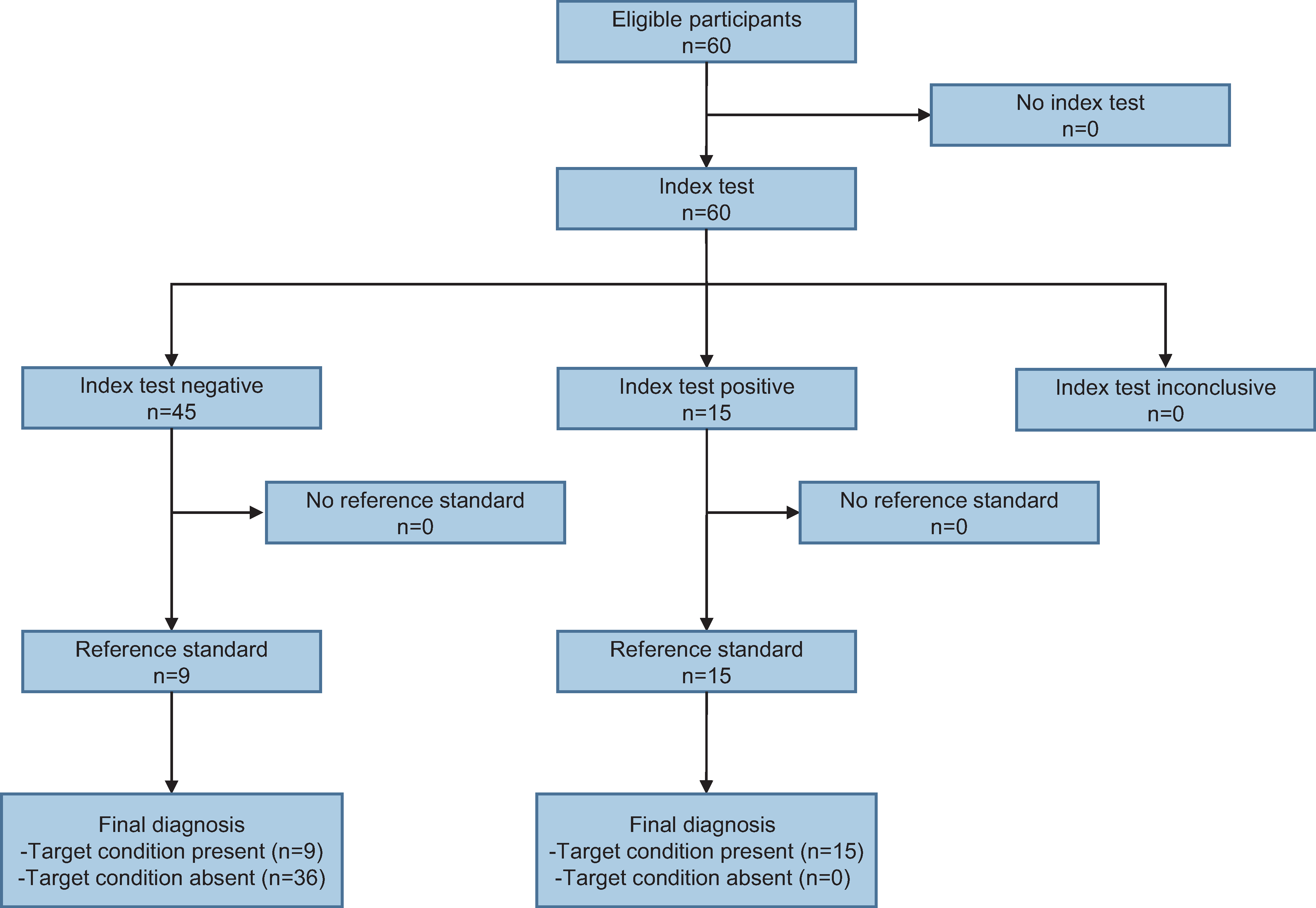

Supplement: S1 Fig — (TIF) [file pntd.0008147.s002.tif]

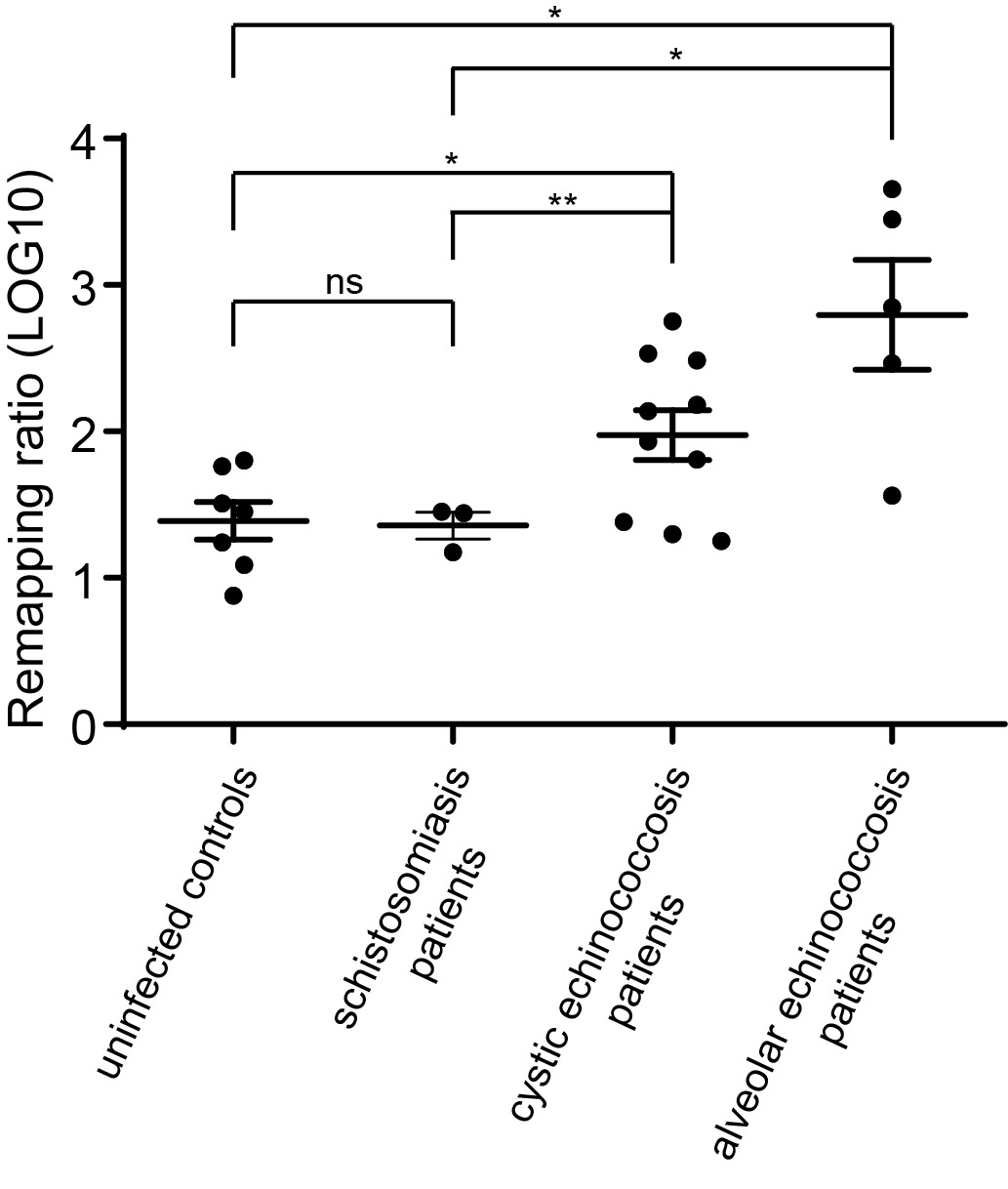

Supplement: S2 Fig — Remapping ratio was transformed by logarithm. E17 and S04 were discarded as outliers because their remapping ratio was zero. The data are expressed as mean ± SEM. * p < 0.05, ** p < 0.01, unpaired t-test with Welch's correction. (TIF) [file pntd.0008147.s003.tif]

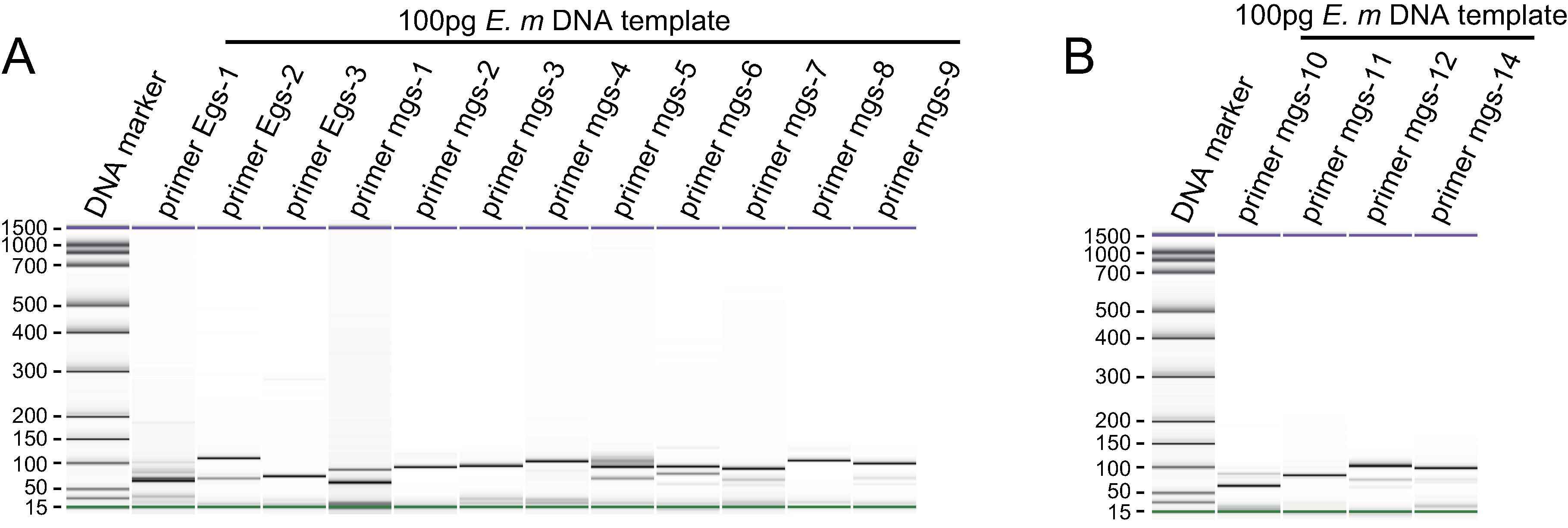

Supplement: S3 Fig — PCR amplification of 100 pg genomic DNA extracted from liver lesion of an alveolar echinococcosis patient in the background of 10 ng human genomic DNA with each pairs of primers. Products of the expected size were observed with primers Egs-2, mgs-1, mgs-2, mgs-3, mgs-4, mgs-5, mgs-6, mgs-7, mgs-8, mgs-9, mgs-11, mgs-12 and mgs-14. The molecular weight (bp) is indicated on the left of each panel. Green lines:15bp. Purple lines: 1500bp. (TIF) [file pntd.0008147.s004.tif]

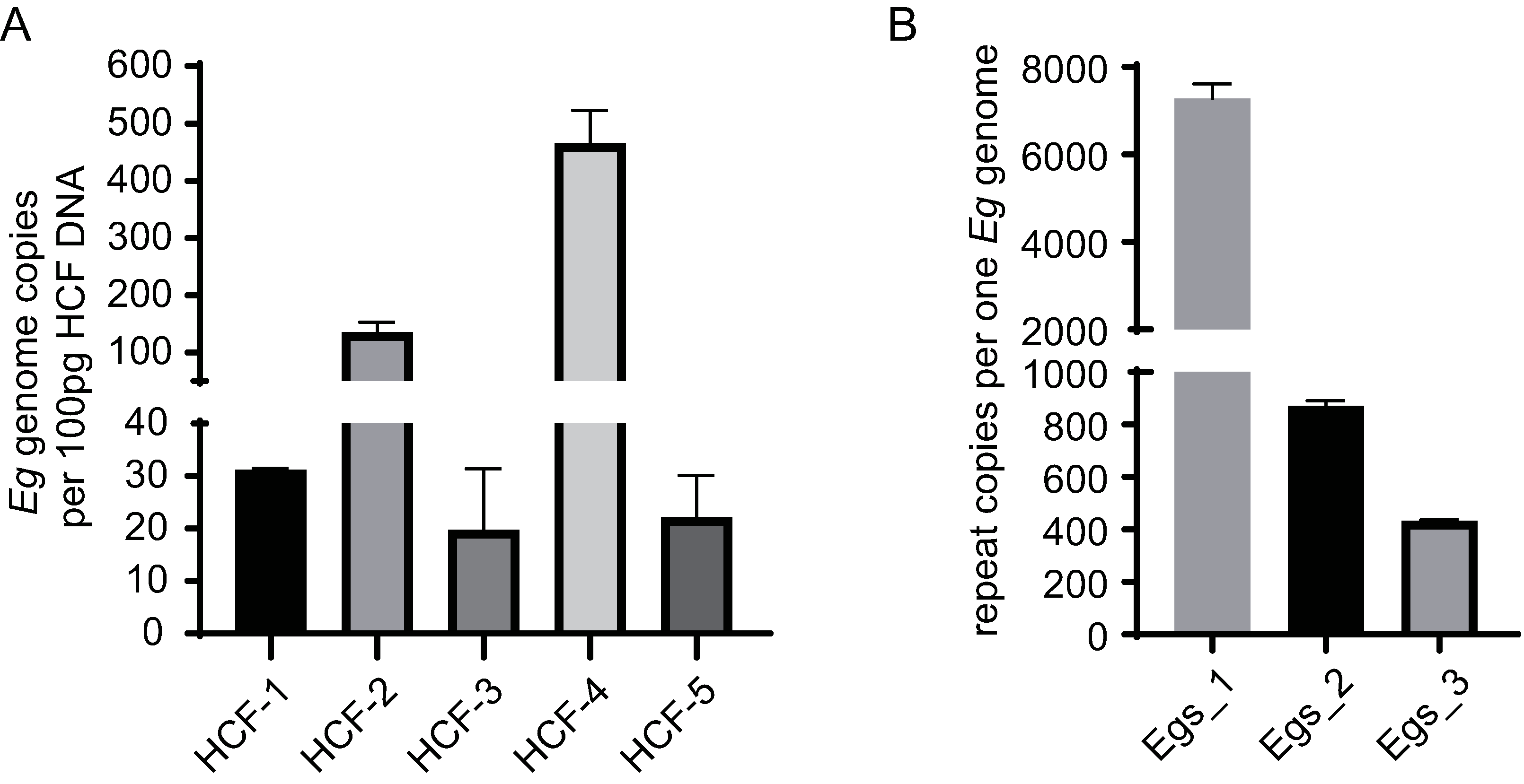

Supplement: S4 Fig — (A) Copy numbers per 100 pg of E. granulosus genome from HCF samples are shown. (B) Copy numbers amplified by indicated 3 pair of primers in one Echinococcus genome. Data is expressed as mean ± SD. (TIF) [file pntd.0008147.s005.tif]
